# Guest Editorial: Symposium breast

**DOI:** 10.4103/0971-3026.50836

**Published:** 2009-05

**Authors:** Bijal Jankharia

**Affiliations:** Consultant Radiologist, Piramal Diagnostic–Jankharia Imaging, Bhaveshwar Vihar, Mumbai, India

I am highly privileged to write the editorial on Breast Imaging.

As we all know, breast imaging has taken a backseat in our country and not many people are actually getting trained in it or pursuing it as a career option in Radiology. Most of the times, a mammography machine is just installed to “complete” the department and be reported by people who report routine X-rays and have sometimes heard a lecture or two on mammography.

However, we do have some nice articles coming in this journal and the next on Breast Imaging. The first one is by Dr. Haydee Ojeda-Fournier who graciously accepted Dr. Linda Olson's as well as my request to write on “MRI for breast cancer: Current indications.” This is an extremely relevant topic as most people today from the patient to the general practitioner, consultant physician, oncosurgeon as well as the radiologist are quite confused as to when a breast MRI should be performed, when it can be performed, and when it need not be performed. This article makes decision making quite clear. The second one is on lupus mastitis and its unusual calcifications, which though uncommon is quite interesting. It is by Dr. Abdul Majid Wani from Saudi Arabia. In fact, I had written an article on the same in 1994–1995 with my teacher Dr. Linda Olson from UCSD, but it never got accepted in the journals that she sent to at that time. The third one is a list of useful websites related to Breast Imaging by Dr. I. K. Indrajeet.

In the forthcoming issue, we have some more articles on breast microcalcifications, breast ultrasound as well as breast tuberculosis. I hope that all of us can get more insight into Breast Imaging after reading them.

There are a few places in Mumbai where students/consultants who are interested in Breast Imaging can get further trained in the same. I would request radiology consultants to get trained for at least a month at one of these centers before they start reporting mammograms, breast ultrasounds or breast MRIs.

Once again, I thank Dr. Bhavin Jankharia and his team to allow me to help them with this and the forthcoming issue on Breast Imaging. It has been quite exciting to do the same.

